# Heterotopic pregnancy following induction of ovulation with clomiphene citrate

**Published:** 2011

**Authors:** Sedigheh Ghandi, Raheleh Ahmadi, Mahmoud Fazel

**Affiliations:** 1 Department of Obstetrics and Gynecology, Sabzevar University of Medical Sciences, Sabzevar, Iran.; 2 Department of Pharmacology, Sabzevar University of Medical Sciences, Sabzevar, Iran.

**Keywords:** *Heterotopic*, *Assisted conception*, *Clomiphene*

## Abstract

**Background:**

Although heterotopic gestation is common in assisted reproductive techniques, it is very rare in natural conception and clomiphene induced pregnancy. Diagnosis and appropriate intervention of heterotopic pregnancy requires a high index of suspicious.

**Case:**

In this paper a case of heterotopic pregnancy in a 30-year old woman with hemoperitoneum from ruptured tubal pregnancy with live intrauterine gestation at 9 weeks of gestation is reported.

**Conclusion:**

This case suggests that a heterotopic pregnancy must always be considered particularly after the induction of ovulation by clomiphene citrate or assisted reproductive technology. Every clinician treating women of reproductive age should keep this diagnosis in mind. It also demonstrates that early diagnosis is essential in order to salvage the intrauterine pregnancy and avoid maternal morbidity and mortality.

## Introduction

Heterotopic pregnancy is defined as the coexistence of intrauterine and extrauterine gestation. The rate of occurrence has been reported to be 1:30.000 pregnancies (1), although a rate of 1:100 has been reported in the assisted reproductive techniques (2). The occurrence of heterotopic pregnancy following induction of ovulation with clomiphene for infertility cases is rare (3, 4).

It will inducea life- threatening complication of pregnancy and its diagnosis is often difficult. When an ectopic pregnancy is suspected following induced ovulation or assisted reproductive techniques, the presence of an intrauterine pregnancy should not reassurantly be considered and the patient should be evaluated rigorously for ruling out the heterotopic pregnancy that can make serious adverse effects on the intrauterine fetus and the mother. 

The aim of this case report is to present a rare case of heterotopic pregnancy following ovulation induction with clomiphene citrate in an anovulatory patient. 

## Case report

A 30 years old woman with a history of early spontaneous abortion conceived after two cycles of ovulation induction with clomiphene citrate. (100 mg/day starting on day 5 of the cycle). She was a case of secondary infertility for one year and ultrasound findings of polycystic ovaries. She had oligomenorrhea and mild hirsutism with normal body mass index. 

Hysterosalpingography showed a normal uterine cavity with spillage of both tubes. The hormonal profile and her husband spermogram were normal. In first cycle of induction ovulation, the dose of 50mg/day was used, but ovulation did not occur. In next cycle the dose of 100mg/day was used. 

In follicular monitoring there were 2 follicles in right ovary measuring 20×18 mm, but pregnancy did not occurred in this cycle. In the second cycle with the same dose, the patient did not return to clinic for sonography. Her next presentation to the clinic was at 6 weeks of gestation, when abdominal ultrasound scan examination revealed an intrauterine pregnancy of corresponding period of gestation and bilateral cystic lesion measuring 35 mm in both ovaries. 

She has no history of pelvic inflammatory disease and was asymptomatic except for nausea and vomiting (hyperemesis gravidarum). Eighteen days later she presented in emergency room with severe acute lower abdominal pain followed by a syncopal attack. 

On examination she was pallor and there was a generalized abdominal distention and tenderness. Ultrasound by a sonologist showed a large amount of free fluid in the peritoneal cavity, a viable intrauterine pregnancy with gestational age of 9wk+3d, a 40mm cystic lesion in left ovary, a 38mm cystic lesion in right ovary and a 33 mm heterogenous mass in relation to the left adnexae. Her hemoglobin was 10 gm/dl, and coagulation parameters were normal. 

On expolatory laparotomy there were approximately 1.5 liter of hemoperitoneum and a ruptured left tubal pregnancy. The uterus was of 8-9weeks size. Left salpingectomy was performed. Pathological examination confirmed a ruptured left tubal pregnancy. Blood transfusion was needed because her hemodynamic was unstable during the surgery. 

**Figure:**
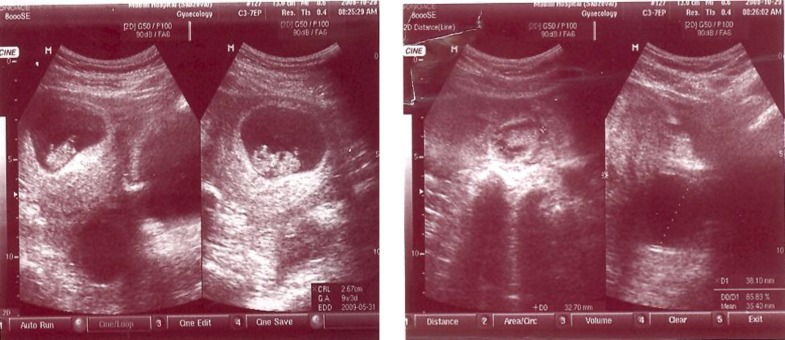


After the operation day, she again complained of severe nausea and vomiting. A repeat ultrasound scan on the third postoperative day showed a viable intrauterine pregnancy. The pregnancy was followed up till term and ended by cesarean delivery with a healthy baby girl.

## Discussion

The case of heterotopic pregnancy following clomiphene induced ovulation was reported in 1971 (5). Clomiphene by hyperstimulation of the ovaries and probably by altering the myoelecrical activity responsible for propulsive action of fallopian tubes, is associated with increased rate of twin and ectopic pregnancy and thus could be associated with a higher rate of heterotopic pregnancy (3, 4). The old traditional teaching that the presence of an intrauterine pregnancy excludes an ectopic pregnancy is not applicable any more in current practice. 

Combined intrauterine and extrauterine pregnancy is a disease with a constant increase in incidence especially in infertile woman subjected to assisted reproductive techniques (3). Heterotopic gestation is difficult to diagnosis clinically. A high suspicion should be present in the managing physician. 

An early diagnosis of heterotopic pregnancy is important for the intrauterine fetus and the mother. Most of the heterotopic pregnancies present in emergency room. Once a heterotopic pregnancy is diagnosed, the next step is how to manage it without producing harms to the intrauterine pregnancy. In an early case, conservative management with laparoscopy or by injecting potassium chloride solution in the ectopic gestational sac under vaginal sonography is advised (6-8).

In Silva case reportheterotopic pregnancy was suspected preoperatively based on transvaginal ultrasonography and the patient was treated laparoscopically by partial salpingectomy and subsequently delivered a normal baby (6). In our case trans abdominal ultrasound was performed after positive pregnancy test. Transvaginal ultrasound is superior to abdominal ultrasound for diagnosis of heterotopic pregnancy. All of the pelvis should be visualized, and the presence of an intrauterine pregnancy should not be reassurant. In Rizk study a total of 20 cases of heterotopic pregnancy resulting from in-vitro fertilization/ embryo transfer at Bourn Hall Clinic were reported. 

The clinical presentation of these cases at first examination was quite variable, with 45% of patients asymptomatic. Tubal pregnancy in one patient resolved spontaneously. Two cases were treated by an injection of potassium chloride into the gestational sac and the remaining 17 cases were treated by salpingectomy. 

In 10 patients the intrauterine pregnancy resulted in live birth and the remaining 10 patients aborted spontaneously (8). In this study (8), patients were undergo assisted reproduction and careful monitoring in early pregnancy by transvaginal ultrasound was performed in all of them. In cases similar to our case with ruptured ectopic pregnancy (9, 10), the standard treatment is surgical removal of the ectopic either by laparoscopy or laparotomy with minimal manipulation of the uterus. 

In Addar case report, heterotopic pregnancy occurred after induction ovulation with combined clomiphene citrate and recombinant FSH. Like our case the next presentation of the patient was at 5 weeks of amenorrhea with severe abdominal pain and vaginal bleeding and in laparotomy, salpingectomy was performed for ruptured tubal pregnancy. 

In Ibha case report, the patient unlike to our case was not asymptomatic and had lower abdominal pain and spotting. Ultrasound scan showed only an intrauterine pregnancy and the patient received human chorionic gonadotropin (hCG). The patient was admitted to emergency room with acute abdominal pain and syncopal attack and on expolatory laparotomy, there were ruptured tubal pregnancy and hemoperitoneum (10). 

The present study like the studies mentioned above emphasizes on the possibility of heterotopic pregnancy following ovulation inducing agents. Because it maybe a life- threatening condition that can be easily missed, we must always exclude the presence of an ectopic gestational sac in the early phase of pregnancy. Knowledge of the possibility of heterotopic pregnancy and its risk factors are important for early diagnosis and avoiding complications. Gynecologists, primary care physicians, sonologists and emergency room physicians should have a high suspicion of heterotopic pregnancy in women conceive after using ovulation inducing agents.
